# Emerging Protein Targets in Triple-Negative Breast Cancer: Beyond Conventional Therapy

**DOI:** 10.3390/cancers18040618

**Published:** 2026-02-13

**Authors:** Andrea Previtali, Isabella Guardamagna, Silvia Calandra, Maryam Shakarami, Leonardo Lonati, Cecilia Riani, Rossella Semerano, Giorgio Baiocco, Maristella Maggi, Claudia Scotti

**Affiliations:** 1 Department of Molecular Medicine, University of Pavia, 27100 Pavia, Italy; andrea.previtali01@universitadipavia.it (A.P.); silvia.calandra01@universitadipavia.it (S.C.); maryam.shakarami01@universitadipavia.it (M.S.); claudia.scotti@unipv.it (C.S.); 2 Department of Physics “A. Volta”, University of Pavia, 27100 Pavia, Italy; isabella.guardamagna@unipv.it (I.G.); leonardo.lonati@unipv.it (L.L.); cecilia.riani01@universitadipavia.it (C.R.); rossella.semerano01@universitadipavia.it (R.S.); giorgio.baiocco@unipv.it (G.B.)

**Keywords:** triple-negative breast cancer, targeted therapy, omics

## Abstract

**Simple Summary:**

Triple-negative breast cancer is one of the most aggressive types of breast cancer and is especially difficult to treat because it does not respond to standard therapies. Although chemotherapy and some immune-based treatments can help certain patients, many tumors eventually stop responding. As a result, there is an urgent need to identify new treatment strategies that are more effective and long-lasting. This review describes recent progress in understanding how triple-negative breast cancer grows and survives, and how this knowledge is being used to develop new therapies. It covers treatments that help the immune system attack cancer cells, drugs that target weaknesses in cancer cell DNA repair, and antibody-based therapies that deliver toxic agents directly to tumors. The review also explains how large-scale molecular studies are revealing new drug targets and how the tissue surrounding the tumor affects treatment success. Finally, it highlights innovative radiation approaches designed to work together with modern drug therapies to improve patient outcomes.

**Abstract:**

Triple-negative breast cancer (TNBC) remains one of the most aggressive and therapeutically challenging breast cancer subtypes, lacking expression of estrogen receptor, progesterone receptor, and HER2. Conventional chemotherapy and immune checkpoint inhibitors provide some benefit, but resistance and relapse are frequent. The search for novel targets has therefore become central to developing more effective and durable therapies. Recent advances in proteomics, structural biology, and targeted protein degradation are rapidly expanding the repertoire of actionable molecules in TNBC. This review summarizes current and emerging therapeutic strategies for TNBC, with a focus on targeted approaches designed to address tumor heterogeneity and resistance mechanisms. To this end, recent advances in targeted therapies are examined, including immune checkpoint inhibitors, PARP inhibitors, Trop-2–directed antibody–drug conjugates, anti-angiogenic agents, PI3K/Akt/mTOR pathway inhibitors, androgen receptor antagonists, and CDK4/6 inhibitors, highlighting results from completed and ongoing clinical trials. In addition, we explore novel targets identified through integrative omics approaches, as well as the role of the tumor metabolism and microenvironment in modulating therapeutic efficacy. Finally, we outline innovative radiotherapy strategies based on targeted radiation delivery and biological integration with systemic therapies. Collectively, this review provides an updated and novel overview of the evolving TNBC therapeutic landscape and highlights promising directions for the development of next-generation, biomarker-driven treatment strategies aimed at improving patient outcomes, maintaining a broad perspective on a very large class of targets.

## 1. Introduction

Breast cancer (BC) is a complex and heterogeneous neoplasia, and it can be classified into different molecular subgroups based on the expression of specific markers, mainly represented by Hormone Receptors (HRs) and Human Epidermal growth factor Receptor-2 (HER2). HRs are the Progesterone receptor (PR) and the Estrogen Receptor (ER). The different phenotypes that derive from the combination of the expression of these cellular markers are classified into the following categories: HR−positive/HER2−negative (HR+/HER2−), which accounts for nearly 70% of the total cases; HER2-positive (HER2+), which represents 15–20% of total BCs; triple-negative breast cancer (TNBC), with a cellular expression of PR and ER of ≤1% and HER2 expression of 0 to 1+ by immunohistochemistry (IHC, according to the American Society of Clinical Oncology/College of American Pathologists ASCO/CAP), which represents 10–20% of the total cases of BC [1,2,3].

Each of the three receptors represents a validated target for BC therapy, and targeted drugs allow a very high therapeutic success, with a survival rate of about 90% at 5 years. For example, for women diagnosed between 2015 and 2021, relative survival is approximately 96% for HR+/HER2−, ~92% for HR+/HER2+ and about 87% for HR−/HER2+ tumors [4]. However, this statistic is much lower, around 78%, for HR−/HER2− TNBC.

This review summarizes the most recent innovative therapeutic approaches and outlines promising directions for developing next-generation therapeutics to address heterogeneity and resistance in TNBC, covering cell surface proteins and receptors, intracellular pathways of interest, and the role of the tumor microenvironment (TME) in current and under-investigation therapies. We will initially focus on completed clinical trials and their results and then move to AI-identified protein targets derived from omics data, metabolic alterations and observations regarding the TME. We will finally summarize the current and innovative radiotherapy (RT) approaches based on the use of radiosensitizers and targeted radiation delivery.

### 1.1. Epidemiology, Risk Factors and Prognosis

TNBC is a common cancer in women, with roughly 2,000,000 cases diagnosed worldwide in 2022 [5]. In fact, it accounts for approximately 10–20% of all breast carcinomas. Moreover, epidemiological data show that TNBC is mostly diagnosed in premenopausal young women under 40 years old [5]. In addition, this tumor appears to be more frequent in African or Hispanic women [2], with African Americans having a worse prognosis compared to other groups [3]. Other non-modifiable risk factors are, as cited above, mutations in *BRCA1* and *BRCA2* genes, as well as *TP53* gene mutations, family history and breast tissue density [3]. Among modifiable risk factors are unhealthy lifestyles and radiation exposure (secondary tumors after radiation therapy, RT) [1,3].

TNBC displays a worse prognosis when compared to the other subtypes of BC. It accounts for 5% of all cancer-caused deaths every year [5]. The median overall survival (OS) reaches 10.2 months with current therapies, and the 5-year survival rate is around 60–65% for regional tumors, and only 11% for metastatic tumors [2,6]. The mortality rate becomes higher in the first 3 months after relapse, reaching 75% [5]. Distant metastases are quite common, occurring in nearly 46% of patients.

### 1.2. Molecular, Histological and Clinical Classification

TNBC is, therefore, characterized by the lack of expression of all three key molecular targets validated for therapy, an aggressive phenotype and an invasive behavior, with genetic instability and mutations in typical genes like Tumor Protein 53 (*TP53*), mutated in >80% of TNBC cases, PhosphatiIdylinositol-4,5-biphosphate 3-Kinase Catalytic subunit Alpha gene (*PIK3CA*) and BReast CAncer gene 1 (*BRCA1*). More than 85% of breast cancers developed following *BRCA1* germline mutations display a triple-negative phenotype [2]. However, the genetic expression, the transcriptional profile and the histological phenotype differ widely [2]. In fact, from a molecular point of view, TNBCs can be classified into the following categories, proposed by Burstein et al. in 2015 [7], based on gene expression/transcriptional profile:

- Basal-Like Immuno Suppressed (BLIS), characterized by downregulation of B, T and natural killer (NK) cell immune-regulating pathways and cytokine pathways;
- Basal-Like Immune Activated (BLIA), displaying an opposite transcriptional profile with respect to the BLIS subtype, with upregulation of genes involved in immune cell activity;
- Mesenchymal (M or MES), enriched for genes involved in cell motility, epithelial–mesenchymal transition (EMT), DNA damage response (DDR) pathways and growth factor pathways such as the Insulin Growth Factor-1 (IGF-1) pathway. This type of cell constitutes metaplastic carcinomas with preferential metastasis to lungs, and shows defects in PhosphoInositide 3-Kinase (PI3K)/protein kinase B (Akt)/mechanistic Target Of Rapamycin (mTOR) (PI3K/Akt/mTOR) pathway;
- Luminal Androgen Receptor (LAR), characterized by Androgen Receptor (AR) signaling and hormonally regulated pathways, including the ER pathway. This feature is due to a small (~1%) subpopulation of LAR TNBC cells that show low ER activation; however, these BCs are still classified as triple-negative because this subpopulation is too small to be detected by immunohistochemistry. This cell type causes a low-grade lobular carcinoma, with increased frequency of lymph node involvement. It represents 11% of all TNBCs [7,8].

Two more categories are also taken into account, according to the transcriptional features of tumor-infiltrating lymphocytes (TILs) and tumor-associated stromal cells:

- Mesenchymal Stem-Like (MSL), which is enriched for stem cell-associated gene expression and angiogenesis gene expression;
- Immuno Modulatory (IM), whose tumor tissue overexpresses immune cell markers, such as Nuclear Factor kappa B (NFKB)*,* Tumor Necrosis Factor (TNF)*,* and Janus Kinase (JAK), and immune regulators, such as Cytotoxic T-Lymphocyte-Associated protein 4 (CTLA4), Programmed Death-1 and Programmed cell Death Ligand-1 (PD-1 and PD-L1), and displays the best prognosis among TNBCs. Rather than a proper tumoral subclass, this phenotype has been defined as the result of an immune modulation of the tumor generated by lymphocyte infiltration in the cancer microenvironment. Moreover, there is evidence that the presence of TILs might be a positive prognostic factor [2,8,9,10,11].

In 2016, Lehmann et al. [8] proposed a novel categorization of TNBCs, keeping the M and LAR classes and adding two more: the Basal-Like 1 (BL1) class and the Basal-Like 2 (BL2) class. The Basal-Like 1 (BL1) class includes ductal, high-grade carcinomas characterized by high expression of genes involved in cell cycle progression and DDR pathways. It displays impairments in Homology Directed Repair (HDR) mechanisms, a characteristic that makes this class sensitive to genotoxic therapies. The Basal-Like 2 (BL2) class is a subgroup of metaplastic carcinoma that largely expresses genes coding for growth factors and for key enzymes of glycolysis and gluconeogenesis [2,8,11]. Along with the M subtype, the BL2 subclass frequently harbors alterations in the PI3K pathway genes (as very often observed in TNBC) and in the Wnt pathway genes, but mutations of β-catenin are remarkably infrequent in these tumors [2]. The BL1 subclass represents 18% of total TNBC cases, while the BL2 class accounts for 13% of TNBCs [8]. Another subgroup of TNBC is the so-called “claudin low,” characterized by low to null expression of luminal differentiation markers, high expression of EMT markers and cancer stem cell–like features, but according to Burstein’s classification, this subgroup is included in the M category [11].

The division in transcriptionally different categories is a tool that can be used to predict the tumor response to different chemotherapeutics, and this helps to stratify patients to provide them with tailored therapy [11]. For example, the BL1 subtype seems to be highly responsive to DNA-targeting drugs like Anthracycline and Cyclophosphamide, followed by Taxane (ACT) (52% of pathological complete response, pCRs), while the BL2 and the LAR subtypes show the opposite effect with 0 and 10% pCRs, respectively. These data highlight the differences between BL1 and BL2 subtypes, even though they are both basal-like tumors [8]. Instead, LAR, M and MSL subtypes are more sensitive to the inhibition of the PI3K/Akt/mTOR pathway [2,8,12]. With regard to the MSL and IM classes, TILs carry a gene expression profile marked by an increased expression of immune checkpoint regulators such as PD1, PD-L1 and CTLA4, which act as molecular targets for therapy [8].

The clinical behavior of TNBC is characterized by aggressiveness, invasiveness and frequent relapses. The outcome of TNBC metastasis is often lethal and involves the brain and visceral organs [2,13]. A high proliferation rate, alterations in DNA repair genes and increased genomic instability are other TNBC typical markers. Histologically, it displays a poorly differentiated phenotype [14].

### 1.3. Current Therapies and Unmet Therapeutic Needs

Due to its low to absent cellular expression of actionable molecular targets, TNBC is not sensitive to endocrine therapy or molecular targeted therapy [6,15]. Therefore, the current therapies include nonspecific chemotherapy (neoadjuvant or adjuvant), surgery and radiotherapy [15].

Chemotherapy is mainly carried out through anthracyclines (e.g., doxorubicin) and taxanes (e.g., paclitaxel), which act to inhibit mitosis and block DNA topoisomerase activity, respectively, in combination with cyclophosphamide and platinum-based drugs (e.g., carboplatin) [16,17]. However, these treatments are associated with severe side effects and resistance, as well as frequent relapse, and these data are confirmed by 70–80% of TNBC patients who do not achieve a complete response [15]. These patients are likely to suffer a recurrence and a lethal tumor metastasis [16]. The treatment of TNBC metastatic disease involves anthracyclines, platinum-based drugs and taxanes, as previously described, as well as capecitabine, gemcitabine, vinorelbine, and eribulin. For tumors carrying *BRCA1/2* germline mutations, monoclonal antibodies conjugated or not with drugs like Sacituzumab Govitecan (SG) or Bevacizumab are implemented. PD-L1-overexpressing cancer patients are treated with atezolizumab or pembrolizumab [16]. However, TNBC is characterized by intrinsic chemoresistance, mainly due to the presence of cancer stem cells (CSCs), ATP-binding cassette (ATP) transporters (e.g., multidrug resistance protein-1 and MRP [10]), hypoxia and avoidance of apoptosis [16].

For radiotherapy (RT), current guidelines do not differentiate irradiation protocols for the various breast cancer subtypes and the molecular subtyping of TNBC has not been integrated with RT protocols yet [18]. Despite this, the role of RT in the treatment of TNBC remains crucial, as described by Haque et al. in 2019 [19]. In this study, out of 8526 TNBC patients older than 70 years, 74% received adjuvant RT and 26% did not. After a median follow-up of 38 months, the 5-year OS was significantly higher in the group that received adjuvant RT (77.2% vs. 55.3%, *p* < 0.001) and this effect was also maintained after stratification for age, stage and chemotherapy implementation [19]. In another comparative study conducted by Abdulkarim et al. [20], locoregional recurrence was reduced with the addition of adjuvant RT in 768 TNBC patients, after a median follow-up of 7.2 years (*p* < 0.01). The American Society of Radiation Oncology published guidelines on the use of radiotherapy after mastectomy, only for patients who failed to achieve a complete nodal response. However, for patients who responded well to neoadjuvant chemotherapy, no definite recommendations were given [18]. Although adjuvant RT can improve the local progression and recurrence of TNBC, there is a percentage of TNBC patients resistant to RT, revealing a radioresistance characteristic in TNBC [18].

The poor prognosis of TNBC patients with the current therapies and the heterogeneity of this tumor drive the research toward new therapeutic strategies.

Here we provide a comprehensive and up-to-date overview of potential novel protein targets for TNBC treatment. Target selection was primarily guided by known tumor vulnerabilities, whose expression levels in TNBC were collected from original research articles and omics databases. To our knowledge, no previous review has integrated such a wide range of proteomic evidence and tumor vulnerability data from both original research and multi-omics databases in the context of TNBC.

## 2. Current Approaches and Clinical Trials

### 2.1. Targeted Therapy in TNBC Patients

The lack of expression of the three known, key extracellular and intracellular receptors for molecules involved in cell growth in TNBC imposes the development of alternative therapeutic strategies. Possible approaches could be targeting immune checkpoints (e.g., PD-1 and PDL-1) by administering Poly ADP-ribose polymerase (PARP) inhibitors (presently used mainly for *BRCA*-mutated TNBCs) or Tumor-associated calcium signal transducer 2 (Trop-2) inhibitors, possibly through antibody–drug conjugates (ADCs), anti-angiogenic agents, PI3K/Akt/mTOR pathway inhibitors, cyclin-dependent kinase (CDK) 4/6 blockers and AR inhibitors for LAR subtypes. The use of the above-mentioned therapeutics is strictly related to the molecular biomarkers of each tumor and to other factors like cancer stage and grade [6,11,21,22].

### 2.2. Current Treatments and Clinical Trials

The phase II clinical trial FASCINATE-N (NCT05582499) serves as a key example of how multiple therapeutic strategies can converge in TNBC. This neoadjuvant precision-based platform assigns patients to distinct treatment arms according to their tumor’s molecular profile, integrating immune-based therapies, DNA damage-targeting agents, and additional pathway-specific treatments. By evaluating diverse targeted approaches within a single platform structure, the study demonstrates how the heterogeneity of TNBC can be addressed through complex, multi-layered therapeutic designs, effectively connecting and reinforcing different therapeutic targets. FASCINATE-N’s goal is to test the efficacy of novel drugs alone or in combination with standard chemotherapy. In particular, patients with different types of TNBC were randomly divided into groups according to molecular typing and subtyping and received targeted treatment or conventional neoadjuvant chemotherapy. As a result, 53 subgroups with specific molecular features were formed. A specific clinical profile could be present in more than one molecularly defined group to test different combinations of drugs, to cover as many therapeutic options as possible in order to determine the best treatment regimens for each subset. The novel agents being tested include CDK 4/6 inhibitors, PD-1/PD-L1 monoclonal antibodies, PARP inhibitors, HER2 inhibitors, Trop-2 inhibitors, and angiogenesis inhibitors. pCR is set as the primary outcome measure; secondary endpoints will be invasive disease-free survival (iDFS), overall/objective response rate (ORR), evaluation of adverse effects using the Common Terminology Criteria for Adverse Events (CTCAE) scale (v 4.0), evaluation of gene expression profile during treatment and number of peripheral blood mononuclear cells (PBMCs) during treatment. The study started in 2022 and is planned to be completed by 2028 [23].

Below is an extensive review of the currently available targeted therapies and ongoing clinical trials on TNBC patients. The ClinicalTrials.gov database [24] was consulted in December 2025. Table 1 summarizes clinical trials investigating targeted therapeutic approaches in triple-negative breast cancer (TNBC). Trials are grouped according to their molecular target and type of intervention and are classified by study status (recruiting, terminated, or withdrawn). For each trial, we report the ClinicalTrials.gov identifier, investigational drug, years of trial development, clinical phase, corresponding citation (when available), and main published outcomes. This comprehensive overview highlights both ongoing efforts and past challenges in advancing targeted therapies for TNBC.

#### 2.2.1. PD-1/PD-L1 Inhibitors

PD-1 is a member of the immunoglobulin superfamily, with a fundamental role in apoptosis. It is a type I transmembrane glycoproteic receptor of 50–55 kDa, with a cytoplasmic tail containing the immune receptor tyrosine-based inhibitory motif (ITIM) and an immune receptor inhibitory tyrosine-based switch motif (ITSM) necessary to its immune function. PD-1 is expressed on mature T cells, B cells and monocytes [40,41]. PD-L1 and PD-L2 are the ligands of PD-1. This ligand–receptor interaction negatively regulates the activity of immune cells, and, when this happens in the TME, it results in an immune suppression against cancer cells. When activated by PD-L1 expressed by tumor cells, in fact, PD-1 brings T cells to apoptosis, and this phenomenon permits tumor cells to evade immune surveillance. Immunotherapy with PD-1 and PD-L1 inhibitors aims to block this interaction and promote T cell activation to generate an antitumor immune effect [41]. PD-1/PD-L1 inhibitors are becoming the standard therapy for various cancers and represent valid tools for immunotherapy [41]. Subtypes of TNBC can be PD-L1-positive (PD-L1+) [42], and, therefore, they are eligible for a therapy based on PD-1/PD-L1 inhibitors. Among these, pembrolizumab and nivolumab (anti-PD-1), as well as atezolizumab, avelumab and durvalumab (anti-PD-L1), are the most well-studied monoclonal antibodies (mAb) for immunotherapy in the last decade.

In TNBC patients, anti-PD-1 mAbs JS001 and PDR001 (spartalizumab) have been demonstrated to be well tolerated during phase I-II clinical trials (NCT02838823 and NCT02404441, respectively). Their antitumor activity, however, has been shown to be moderate [43,44]. Pembrolizumab is a humanized mAb with high affinity for PD-1. It has been commercialized under the name Keytruda since 2017 and, after years of clinical trials, it is currently used in the treatment of melanoma, non-small cell lung cancer (NSCLC), Merkel cell carcinoma and lymphomas. The Food and Drug Administration (FDA) approved pembrolizumab for advanced and metastatic TNBC treatment in 2020, and this regimen is now the standard of care first-line treatment for PD-L1+ TNBC patients [45].

Several clinical trials using anti-PD-L1 mAbs in TNBC are ongoing. Among them, the phase III clinical trial TROPION-Breast05 (ClinicalTrials.gov ID: NCT06103864) challenges pembrolizumab efficacy, testing the anti-tumoral effects of datopotamab deruxtecan (Dato-DXd), an anti Trop-2 mAb (see below), with or without durvalumab (Imfinzi, already in use for urothelial carcinoma and NSCLC, [46]) in comparison with pembrolizumab [25].

Atezolizumab (commercialized as Tecentriq), currently used in the treatment of urothelial carcinoma, NSCLC, small cell lung cancer SCLC, hepatocellular carcinoma HCC and melanoma, and avelumab (Bavencio), already in use in the treatment of Merkel cell carcinoma and metastatic urothelial carcinoma [47], are two anti-PD-L1 mAbs under investigation in TNBC patients in the phase I clinical trials NCT03170960 and NCT04360941 (PAveMenT), respectively. The main goal of these studies is, besides determining safety, to assess the efficacy of these mAbs. The study on atezolizumab is expanded to 18 cohort-specific tumors, and the first results on urothelial, gastric, prostate and renal cancer have already been published [47,48,49,50].

Three clinical trials on PD-1/PD-L1 inhibitors were terminated without any specific outcome (NCT02936102, NCT04916002, NCT03549000).

#### 2.2.2. PARP Inhibitors

In recent years, PARP inhibitors have emerged as an important treatment option and are now considered part of the standard of care for TNBC patients carrying germline *BRCA1/2* mutations [51]. Although numerous agents are currently under investigation as potential treatments for TNBC, the PARP inhibitors olaparib and talazoparib remain the most clinically validated and widely adopted options [52,53]. The mechanism can be summarized as follows: beyond simply inhibiting PARP’s enzymatic function, PARP inhibitors can also trap PARP proteins on DNA, forming cytotoxic PARP–DNA complexes. Importantly, the ability to trap PARP does not correlate with catalytic inhibition and differs markedly among available PARP inhibitors. Preclinical studies suggest that this trapping effect may contribute more to antitumor activity than enzymatic inhibition alone [51].

Veliparib (NCT01306032), olaparib (DORA, NCT03167619 and NCT00679783), rucaparib (BRE09-146, NCT01074970), niraparib (TOPACIO/KEYNOTE-162, NCT02657889), and talazoparib (BMN-673, NCT02401347) were tested in phase II clinical trials in TNBC patients.

Veliparib was demonstrated to be well tolerated and showed promising activity in a subset of patients with *BRCA* mutations (NCT01306032; [54,55]), as did niraparib when administered in combination with pembrolizumab (TOPACIO/KEYNOTE-162, NCT02657889; [56]) in terms of objective response rate (ORR) and disease control rate (DCR). Promising results and good tolerability were also observed after administrating olaparib in combination with durvalumab in advanced or metastatic TNBC patients (DORA, NCT03167619; [57]). The combination of olaparib and the heat shock protein 90 (HSP90) inhibitor onalespib (phase I clinical trial, NCT02898207) showed preliminary evidence of antitumor activity. However, single agent administration of olaparib failed to provide successful efficacy [58]. On the contrary, the outcome of a clinical trial on talazoparib as a single agent in HER2-negative BC without germline mutations in *BRCA1/2*, but with either homologous–recombination pathway gene mutations (e.g., Partner and Localizer of BRCA2 *PALB2*, *CHEK2*, etc.) or high tumor homologous recombination deficiency (HRD) scores showed an interesting antitumor effects in terms of partial response and clinical benefit rate (CBR, [59]). These data suggest that talazoparib can be active in a subset of advanced TNBC/HER2-negative breast cancers even in the absence of *BRCA1/2* mutations, provided that other HR-pathway defects are present. Finally, the combination of rucaparib with canonical cisplatin did not significantly improve 2-year DFS or 5-year DFS in TNBC patients [60].

Pamiparib (BGB-290) underwent a phase I trial (NCT03150810) in combination with the alkylating agent temozolomide (TMZ) in patients with locally advanced or metastatic solid tumors, including those with advanced or metastatic TNBC. The study demonstrated that pamiparib combined with low-dose TMZ is feasible and tolerable in the heavily pretreated metastatic/advanced solid-tumor population and shows modest antitumor activity; benefits appear to be more likely in tumors with HR-repair deficiency rather than in unselected cases [61].

Two recruiting trials illustrate complementary strategies being tested in early- and advanced-stage TNBC settings to improve outcomes for breast cancer patients through biomarker-driven intensification and novel agents. The randomized phase II-III study (NCT03150576), in which the PARP inhibitor olaparib is combined with a platinum–taxane backbone, showed an increased pCR and an improvement in long-term outcomes in TNBC and/or germline *BRCA*-mutant patients. An early-phase clinical trial (NCT05933265) using LP-184 in refractory disease showed good results as a first-in-human dose-finding and safety study, and preliminary data show augmented activity in tumors with DNA damage repair defects. While not limited to breast cancer, its focus on pharmacokinetics, tolerability, and exploratory biomarker correlations provides evidence that LP-184 could, if active and tolerable, be considered as a later-line option for patients who progress after standard and targeted neoadjuvant/adjuvant therapies or could offer mechanistic insight relevant to PARP-sensitive biology.

It is worth mentioning that between 2015 and 2024, several early-phase clinical trials investigating PARP inhibitors in combination with other agents were terminated before completion, primarily due to issues related to funding, slow enrollment, or sponsor decisions (NCT03875313 on the glutaminase inhibitor CB-839 in combination with talazoparib; NCT03801369 on olaparib combined with durvalumab, selumetinib, capivasertib or ceralasertib in TNBC; NCT04916002 on the effects of cemiplimab with vidutolimod in advanced cancers; NCT05252390, which evaluated NUV-868 alone and in combination with olaparib or enzalutamide; NCT02419495, which assessed selinexor combined with multiple chemotherapy or immunotherapy regimens in advanced malignancies).

#### 2.2.3. Trop-2-Targeted Antibodies

Trop-2 is a transmembrane glycoprotein that was initially observed in trophoblast and fetal tissues. Subsequent studies have demonstrated its overexpression in a wide range of solid tumors, such as breast cancer. This cancer-associated overexpression, in contrast to limited expression in the normal condition in adult tissues, highlights the rationale for targeting Trop-2 therapeutically. Currently, there are many new approaches related to Trop-2-targeted ADCs for breast cancer treatment. ADCs [62] are molecules composed of a monoclonal antibody directed against a tumor-associated antigen, linked via a chemical bound to a cytotoxic payload. Upon binding to the antigen on cancer cells, the ADC is internalized, and the toxic payload is released, causing cell death with the aim of maximizing tumor killing while minimizing damage to normal tissues and overcoming some of the limitations of conventional chemotherapy in TNBC [63,64,65].

Two important molecules of this category are Sacituzumab Govitecan (SG) and Dato-DXd, which showed antitumor activity. Thus, there are many new approaches in which Trop2-directed ADCs are combined with other forms of therapy, including immunotherapy, targeted therapies, and small molecule inhibitors [66,67,68]. The ASPRIA trial (NCT04434040), for example, combines SG and atezolizumab to target minimal residual disease detectable through circulating tumor DNA. SG is the protagonist of the phase I/II basket trial NCT01631552, which evaluated SG combined with the active metabolite of irinotecan (SN-38) in patients with metastatic TNBC who had already received at least two prior systemic therapies for metastatic disease. Overall, this study provided early evidence that SG has clinically meaningful activity in heavily pretreated metastatic TNBC, justifying further development, evidence which later supported regulatory approval of the drug in this indication. The toxicity profile of SG in this study was considered manageable and consistent with its mechanism [69,70,71].

Several ongoing clinical trials are actively exploring Trop-2-targeted ADCs as a promising therapeutic approach for advanced or metastatic TNBC either as monotherapy or in combination with immunotherapy or other targeted modalities, aiming to expand options for patients who have already received treatments. The NCT05749588 and NCT06649331 trials, for example, investigate a Trop-2-directed ADC alone or combined with immunotherapy, assessing objective response rates across different TNBC molecular subtypes, while also monitoring progression-free survival (PFS) and overall survival (OS), duration of response (DoR), disease control and safety. Similarly, NCT06851299 explores multiple therapeutic strategies, including ADC monotherapy, ADC with immunotherapy, and a triple combination with anti-angiogenic therapy, using Topoisomerase 1 (TOP1) inhibitor–based ADCs, to determine both clinical efficacy and safety across diverse patient cohorts.

Similarly, NCT06841354 is testing the efficacy of sacituzumab tirumotecan, either alone or in combination with pembrolizumab, versus standard chemotherapy in PD-L1–low TNBC patients.

Other adaptive platform clinical studies, such as NCT06649331, employ Bayesian response-adaptive designs to evaluate rechallenge with Trop-2, HER2, HER3, and Nectin-4 ADCs in heavily pretreated metastatic breast cancer. These trials seek to identify the most effective regimens based on objective response, molecular signatures, and PFS, while continuously optimizing the study arms for maximum benefit. Single-arm studies, like NCT06793332, are exploring the combination of bispecific antibodies, such as ivonescimab (AK112), with Trop-2 ADCs, particularly in patients with brain metastases, with the dual goals of confirming safety and demonstrating both intracranial and extracranial antitumor activity in populations with a historically poor prognosis.

Innovative imaging strategies are also being integrated into the evaluation of Trop-2 ADCs. The exploratory trial NCT07046455 uses the positron emission tomography (PET) probe 89Zr-DFO-hSR7 to monitor Trop-2 expression and treatment response in patients receiving SG, correlating imaging signals with tissue expression and clinical outcomes, while also exploring mechanisms of resistance and biodistribution. Complementing these efforts, multicenter studies such as NCT06878625 assess Trop-2 ADCs in combination with PD-1 blockade, with or without additional anti-angiogenic therapy, focusing primarily on PFS while incorporating extensive biomarker and safety analyses.

Several pivotal trials are establishing the role of Trop-2 ADCs in both metastatic and neoadjuvant settings. NCT06841354 evaluates sacituzumab tirumotecan, alone or combined with pembrolizumab, in previously untreated, unresectable or metastatic TNBC, with endpoints including overall survival and PFS, objective response, and DoR.

Dato-DXd, mentioned before, is also being investigated in multiple trials, including NCT06974604 and the already cited TROPION-Breast05 (NCT06103864).

Together, these trials represent a concerted effort to define the therapeutic potential of Trop2-targeted ADCs across different clinical contexts, from heavily pretreated metastatic disease to neoadjuvant therapy, and from monotherapy to rationally designed combinations, reflecting the growing emphasis on precision medicine and biomarker-driven treatment strategies in TNBC.

Only one trial (NCT03901469) was prematurely terminated due to the fact that an interim analysis indicated insufficient efficacy, with no safety issues involved. This trial investigated the combination of ZEN003694, a BET inhibitor, with talazoparib, a PARP inhibitor, in TNBC patients who were *BRCA* wild-type, including those who had previously received Trop-2 ADCs. While Trop-2 ADCs directly deliver cytotoxic payloads to Trop-2 expressing cells, the ZEN003694 plus talazoparib combination exploited epigenetic modulation and DNA repair inhibition, potentially overcoming resistance mechanisms and expanding therapeutic options for TNBC.

#### 2.2.4. Anti-Angiogenic Agents

Angiogenesis is the process of developing new immature and disorganized blood vessels within tumorigenesis that let cancer cells obtain the oxygen and nutrients they need to proliferate. The expansion of the tumor increases oxygen consumption and generates regions of the malignant mass that lie far from blood vessels, leading these areas to experience hypoxic conditions. This crucial step in tumor development is called the angiogenic switch. Hypoxia triggers the nuclear translocation of Hypoxia-Inducible Factors (HIFs) in cancer cells. HIF-1α dimerizes with HIF-1β and the complex binds the hypoxia response element (HRE) on the DNA, acting as a transcriptional factor for pro-angiogenic mediators like Vascular Endothelial Growth Factor (VEGF), Placental Growth Factor (PlGF), Fibroblast Growth Factor (FGF) and Platelet-Derived Growth Factor (PDGF). When these factors reach their receptors on tip and stalk cells, specialized endothelial cells that emerge during angiogenesis to form sprouts, their activated pathway crosstalk and determine the sprouting of blood vessels toward the hypoxic regions of the tumor. The persistent and deregulated presence of pro-angiogenic factors marks a difference with the normal genesis of arteries, veins and capillaries, and causes the formation of abnormal, disorganized and chaotic vessels: tumor-induced vessels, in fact, are characterized by heterogenous size and caliber and irregular blood flow, which fail to provide oxygen and nutrients to all cancer cells, leaving hypoxic areas. The abnormal structure of the tumor vessels and the impaired blood perfusion block the infiltration of immune cells, which creates an immunosuppressive tumor microenvironment, and interferes with drug delivery. Therefore, in a counterintuitive approach, targeting angiogenesis to restore a normal blood flow in the TME would permit the perfusion of drugs and immune cells in the microenvironment [72,73,74].

Bevacizumab, commercialized as Avastin and used to treat metastatic breast cancer, NSCLC, glioblastoma, renal cell carcinoma, ovarian cancer and cervical cancer, is an anti-VEGF mAb (32335505). Its safety and efficacy in TNBC have been tested in phase II clinical trials NCT03577743 (RIBBON-2) and NCT06817525. While the first one showed positive results (22415477), the outcomes of the latter have not been published yet.

Other anti-angiogenic agents are under investigation. Lenvatinib (commercialized as Lenvima) is a powerful anti-angiogenic agent, a tyrosine kinase inhibitor, already in use to treat medullary, anaplastic thyroid, adenoid cystic and endometrial cancer [75]. The phase II clinical trial MK-7902-005/E7080-G000-224/LEAP-005 (NCT03797326) tested its efficacy and safety in combination with anti-PD-1 mAb Pembrolizumab in a cohort of patients with solid tumors, including TNBC, and demonstrated antitumor activity with a manageable safety profile [76].

Another tyrosine kinase inhibitor, an anti-angiogenic agent is anlotinib (commercial name FOCUS V, AL3818), already approved by China Food and Drug Administration (CFDA) for the treatment of NSCLC [77]. Its efficacy in combination with anti-PD-1 mAb sintilimab on TNBC is under investigation in the context of the phase II clinical trial NeoSACT (NCT04877821). The combined treatment exhibited a very rapid response, with 96.6% of patients achieving tumor reduction by ≥30% after just one cycle of treatment [36].

An innovative anti-angiogenic molecule is B1962, a bispecific antibody against PD-L1 and VEGF with a high anti-angiogenic activity, which is being studied in a phase II clinical trial NCT06724263, after phase I demonstrated excellent safety and promising therapeutic effects.

#### 2.2.5. PI3K/Akt/mTOR Pathway Inhibitors

Among the multiple molecular pathways activated in TNBC, the PI3K/Akt/mTOR signaling pathway plays a fundamental role in protein synthesis, cell proliferation and survival. Its deregulated activation in TNBC (25% of cases) leads to increased cell proliferation and abnormal cell behavior. Therefore, it is considered a promising molecular target for TNBC treatment [78,79].

Buparlisib (BKM120), PQR309 and gedatolisib (PF-05212384) are three different PI3K inhibitors which, in their clinical trials (NCT01629615, PIQHASSO NCT02723877 and NCT01920061, respectively), displayed different results. Buparlisib, despite its molecular effects on PI3K, may not be sufficient as a single agent in TNBC [80], while its use in combination with gedatolisib resulted in dose-limiting toxicities in 10% of patients and a complete response in one patient in each TNBC arm [81].

In the context of PI3K inhibitors, several clinical trials are ongoing using different molecules. BCTOP-T-M03 reached a phase III clinical study (NCT05954442) on anti-tumoral activity and effects on quality of life. Tenalisib (δ- and γ-PI3K specific inhibitor) and MEN1611 (selective inhibitor of α- β- and γ-PI3K, with reduced activity on the δ isoform) are being tested in phase II clinical trials (NCT06189209 and SABINA NCT05810870, respectively), in which safety and efficacy are the main endpoints.

The safety, tolerability and efficacy of β-PI3K inhibitor AZD8186 on TNBC are being tested in a phase I clinical study (NCT03218826). The first results showed that the maximum tolerated dose (MTD) was not reached and the most frequent adverse events were anemia (57%), diarrhea (43%) and fatigue (43%) [37].

Four clinical trials on PI3K inhibitors have been terminated, of which two were due to excessive toxicity of the tested compounds (GDC-094 NCT01918306 and taselisib NCT02457910). Studies no. NCT04216472 and NCT02476955 were terminated; the first one was due to low participation.

#### 2.2.6. Androgen Receptor Inhibitors

The AR is expressed in many human tissues, including BC tissue. In fact, it has been noted that 70–90% of BC patients overexpress AR, making this marker a possible drug target. Concerning TNBC, the LAR subtype is characterized by AR expression and, considering the lowest rate of pCR to chemotherapy (21.4% vs. that of of BL TNBCs at 65.6%), this can be used as a prognostic factor [82,83]. When androgens (testosterone, T, or dihydrotestosterone, DHT) enter the cells and bind their receptor located in the cytoplasm, the complex moves to the nucleus and binds to the Androgen Responsive Element (ARE) on the DNA. This results in a change in gene expression altering apoptosis, differentiation, angiogenesis and proliferation. There is an interesting crosstalk between AR and PI3K pathways, supported by the fact that, in TNBC, the frequency of PIK3CA mutations in AR+ tumors seems higher than that in AR-negative tumors. Androgen signaling upregulates Phosphatase and TENsin homolog (PTEN) expression. PTEN is PI3K’s main counterpart, as it dephosphorylates phosphatidyl-inositol-3,4,5-triphosphate (PIP_3_) in phosphatidyl-inositol-4,5-biphosphate (PIP_2_). The activation of PTEN restrains PI3K action, which weakens AR activity. In TNBC, AR and PI3K overexpression is often paired, and in these cases the abundance of PI3K bypasses AR-mediated PTEN upregulation. Therefore, targeting only AR will result in a lack of support of PTEN activity, paving the way for PI3K proliferative activity. Hence, dual targeting of the AR and PI3K may produce a synergistic antitumor effect [84].

Bicalutamide (commercialized as Casodex and used in the treatment of prostate cancer [85]) and darolutamide (Nubeqa, also a prostate cancer drug [86]) are two AR antagonists whose tolerability and anti-cancer effects are being tackled in clinical trials NCT03090165 and NCT07016399, respectively.

#### 2.2.7. CDK 4/6 Inhibitors

Cyclin-dependent kinases (CDKs) are the primary enzymes regulating cell-cycle progression. Their activation depends on binding with specific cyclins. Specifically, CDK 4/6 acts as a critical regulator of the G1/S transition. In this process, Cyclin D1 binds to CDK 4 and 6; once this complex is activated by CDK-activating kinases (CAKs), it phosphorylates the tumor suppressor Retinoblastoma protein (Rb). In its dephosphorylated state, Rb binds to and inhibits the E2F transcription factor. However, phosphorylation by CDK 4/6 releases E2F, which then promotes the expression of DNA synthesis genes and Cyclin E, driving the cell cycle forward. Dysregulation of this pathway causes the uncontrolled proliferation characteristic of cancer. Consequently, inhibiting CDK 4/6 can arrest the cell cycle in the G1 phase, effectively blocking tumor growth.

In TNBC, targeting this pathway is complicated by frequent Rb dysfunction, occurring in approximately 30% of cases [87,88,89,90]. While TNBC cell lines exhibit sensitivity to CDK 4/6 inhibition, they are generally less responsive than ER+ lines [91]. This partial resistance is often attributed to the loss of Rb (seen in 7–20% of TNBC) and the overexpression of Cyclin E [88].

Several CDK inhibitors are currently under clinical investigation for TNBC. Trilaciclib is a CDK 4/6 inhibitor primarily used to mitigate chemotherapy-induced bone marrow suppression [92]. Its efficacy in TNBC combination therapy was evaluated in phase II (NCT05112536) and phase III (PRESERVE 2, NCT04799249) trials. Phase II data suggested a positive correlation between favorable outcomes and higher levels of tumor-infiltrating lymphocytes (TILs), specifically a high CD8+/T regulatory cell ratio. Phase III results are currently undergoing quality control review [93].

Ribociclib and Palbociclib are two CDK 4/6 inhibitors under investigation in phase I and II clinical trials named CHARGE (NCT04315233) and CAREGIVER (NCT05067530), respectively. Although already FDA-approved for HR+ [94] and HR+/HER2− breast cancer [95], respectively, Ribociclib (Kisqali) and Palbociclib (Ibrance) are being investigated for TNBC in the CHARGE (NCT04315233) and CAREGIVER (NCT05067530) trials. Emerging studies are also exploring CDK2 as a target. Two molecules, AVZO-021 (NCT05867251) and NKT3964 (NCT06586957), are currently in phase I/II trials to assess their safety and pharmacokinetics in solid tumors, including TNBC. Notably, while four clinical trials involving CDK 4/6 inhibitors were recently terminated, these closures were not due to concerns regarding safety or efficacy [92].

## 3. New Possible Targets Identified by Omics Approaches

Deposited omics data yield unique TNBC marker combinations, with integrative analyses spanning transcriptomics, genomics, proteomics, epigenomics, and, in one instance, metabolomics identifying cancer-specific signatures. Across deposited omics studies, predictive performance metrics refer either to the ability of multi-marker signatures to classify triple-negative breast cancer samples from other breast cancer subtypes or normal tissue, or to stratify TNBC patients according to survival outcomes. Unless otherwise specified, reported performance metrics were extracted from the original studies and obtained using cross-validation or independent validation cohorts based on public datasets such as TCGA and GEO. In this context, gene panels ranging from three to 100 markers achieve classification accuracies of up to 98.9% for TNBC discrimination, while survival-oriented models report concordance indices up to 0.7573; additionally, a 29-protein signature reached 88% accuracy in distinguishing TNBC from luminal tumors and showed strong correlations with patient survival. Validation approaches remain heterogeneous and include experimental methods (cell lines, CRISPR screens, Western blot) as well as in silico analyses (public datasets, survival curves).

To extract the most significant proteins and targets from these datasets, we used the Elicit AI research assistant to perform a literature search and identify candidates based on semantic similarity to the query: “Among the deposited omics data, is it possible to identify unique combinations of markers for triple-negative breast cancer? Select also targets which are not known to be drugged,” across over 138 million academic papers indexed by Elicit, including Semantic Scholar and OpenAlex. The AI retrieved a list of potentially relevant proteins, which were then manually checked one by one to determine whether they had been previously studied in TNBC, with their relevance and novelty discussed individually. Additional proteins not retrieved by Elicit were incorporated through manual literature searches. The final table summarizes only those targets supported by explicit evidence in the primary literature.

Among the molecules that this approach identified as associated with TNBC pathogenesis and progression are nine proteins: GGH, TYMS, PTK6, TOP1MT, SMO, CSF1R, EPHB3, TRIB1, and LAD1 (Table 2 and Table 3). These proteins span diverse functional categories, including nucleotide metabolism, DNA topology, kinase signaling, developmental pathways, and cytoskeletal interactions. Notably, several of them are implicated in cell proliferation, apoptosis resistance, immune modulation, and metastatic behavior, which represent hallmarks of aggressive TNBC phenotypes.

### 3.1. γ-Glutamyl Hydrolase (GGH)

The key function of γ-Glutamyl Hydrolase (GGH), an enzyme mainly located in the lysosome, is to eliminate polyglutamate residues from folates and antifolates. GGH can then be released into the extracellular space, where it maintains its enzymatic activity [103,104] (UniProt: Q92820). It has an effect on DNA methylation, synthesis, and repair. GGH has been found to be a significant molecular marker in a number of cancers, such as pancreatic adenocarcinoma, lung adenocarcinoma, and kidney renal clear cell carcinoma [105]. GGH is found in tumor tissues in TNBC, indicating a possible function in promoting fast proliferation via folate metabolism [106,107]. It has been demonstrated that GGH regulation influences the chemosensitivity of cancer cells to antifolates like methotrexate and 5-fluorouracil, probably by changing the intracellular absorption of drugs and folate polyglutamate concentrations [108]. Although increased GGH expression in breast cancer has been linked to poor clinical outcomes, its particular prognostic function in TNBC remains unclear [107].

### 3.2. ThYMidylate Synthase (TYMS)

By catalyzing the reductive methylation of dUMP to dTMP using 5,10-methylenetetrahydrofolate as a one-carbon donor, ThYMidylate Synthase (TYMS) plays a crucial part in nucleotide metabolism. TYMS promotes both cytosolic and de novo mitochondrial thymidylate synthesis pathways via its presence in the nucleus, cytoplasm, and several mitochondrial compartments, including the matrix and inner membrane [109,110] (UniProt: P04818). Increased proliferation, improved DNA repair ability, and resistance to antifolate-related chemotherapies are often linked to the expression of TYMS in malignancies [111,112,113,114]. In a number of cancers, such as kidney renal clear cell carcinoma, liver hepatocellular carcinoma, lung adenocarcinoma, and pancreatic adenocarcinoma, increased levels of TYMS have been discovered to be prognostic biomarkers related to a poor survival outcomes [105,111,112,113]. Preclinical models have demonstrated that high TYMS levels in TNBC are associated with a poor prognosis, a dedifferentiated tumor phenotype, and a reduction in response to thymidylate-pathway-targeting therapies [115,116,117].

### 3.3. Protein-Tyrosine Kinase 6 (PTK6)

Protein-tyrosine kinase 6 (PTK6) is a non-receptor tyrosine kinase that is a member of the Tyr protein kinase family’s BRK/PTK6/SIK subfamily [118,119] (UniProt: Q13882). It is also referred to as breast tumor kinase (BRK) [118,119] (UniProt: Q13882). This enzyme regulates a number of signaling pathways that control the development of tumors as well as the differentiation and survival of healthy epithelial tissues [119,120]. PTK6’s biological impacts are extremely context-specific and differ based on the type of cell and its subcellular location. For example, it may stimulate apoptosis or differentiation when it is located in the nucleus, but it more frequently promotes oncogenic signaling when it is located in the cytoplasm [120,121]. The RNA-binding proteins SAM68, SLM1, SLM2, and PSF; transcription factors STAT3 and STAT5; and signaling molecules p190RhoGAP, paxillin, BTK/AKT, and STAP2 are among the many substrates of PTK6 that have been found [121,122]. PTK6 has been demonstrated to enhance metastasis and tumor cells’ survival in TNBC. It is increased in response to low oxygen (hypoxia) and glucocorticoid-induced stress through an HIF/GR/PELP1 signaling network [123]. Furthermore, PTK6 suppression in TNBC cell lines decreases migration and increases E-cadherin levels by stimulating the proteasomal degradation of the EMT driver SNAIL [124]. Furthermore, RhoA and the Aryl hydrocarbon Receptor (AhR) seem to be activated by the SH2 domain of PTK6 in TNBC, which drives both cell mobility and paclitaxel resistance [119]. Clinically, TNBC patients with high PTK6 expression levels have decreased survival rates over time and lymph node metastasis, indicating PTK6 as a possible diagnostic marker and therapeutic target [124,125].

### 3.4. Mitochondrial DNA Topoisomerase I (TOP1MT)

TOP1MT (mitochondrial DNA topoisomerase I), a kind of IB topoisomerase, reduces supercoiling and twisting contraction in mtDNA by partially cleaving and reassembling one strand of the DNA double helix (UniProt: Q969P6) [126]. Its catalytic tyrosine creates a covalent DNA–(3′-phosphotyrosyl) intermediate, facilitating strand rotation and potential religation despite a requirement for ATP (UniProt: Q969P6) [126]. Divalent metal ions like Ca^2+^ or Mg^2+^ are necessary for this enzymatic process (UniProt: Q969P6) [126]. TOP1MT is widely expressed, but its RNA quantities are especially significant in tissues that require a lot of energy, like the heart, brain, skeletal muscle, and fetal liver (UniProt: Q969P6) [126]. Because its loss alters the topology of mtDNA and reduces the expression of mitochondrial genes, TOP1MT is essential for the maintenance of mitochondrial DNA [127]. TOP1MT dysregulation has been connected to tumor formation; either high expression or inadequate expression could contribute to the development of cancer, possibly via impacts on DNA damage, oxidative stress, and mitochondrial function [128]. Furthermore, in some tumors, such as liver hepatocellular carcinoma and renal carcinomas, a high TOP1MT level is linked to a poor prognosis [128]. Decreased mtDNA content, compromised respiration in the mitochondria, and a change in metabolic rate toward glycolysis are common characteristics of severe TNBC tumors [129]. While this data does not appear to directly link to TOP1MT, it suggests that mitochondrial–maintenance enzymes like TOP1MT might have a significant role in TNBC pathophysiology.

### 3.5. Smoothened Receptor (SMO)

The SMOothened receptor (SMO) is a multi-pass cell-surface G-protein-coupled receptor that mediates the Hedgehog signaling pathway, regulating cell fate, proliferation and tissue patterning. The Hedgehog (Hh) pathway is frequently dysregulated in TNBC, with overexpression of SHH, PTCH1, SMO, and GLI genes correlating with higher grade and worse prognosis [96]. In TNBC, Hh signaling promotes proliferation, invasion, and especially CSC maintenance, contributing to chemoresistance [96]. Importantly, SMO can mediate both autocrine and paracrine Hh activation: TNBC cells often secrete Hh ligands, whereas SMO-positive stromal fibroblasts respond by remodeling the TME and supporting CSC niches [130]. SMO inhibition in preclinical TNBC models reduces CSC markers, suppresses invasion, and sensitizes tumors to taxanes [130]. Clinically, the SMO inhibitor sonidegib combined with docetaxel demonstrated preliminary benefit in the EDALINE phase I trial, with a subset of metastatic TNBC patients achieving clinical responses [130]. However, some basal-like/TNBC subtypes activate GLI transcription factors independently of SMO—for example via FOXC1—suggesting intrinsic resistance to SMO-targeted therapy [131]. Despite these limitations, SMO remains a promising target in TNBC subsets characterized by ligand-dependent, stromal-driven Hh activation, supporting further development of biomarker-guided SMO-based combination therapies [130,131].

### 3.6. Colony-Stimulating Factor 1receptor (CSF1R)

CSF1R is a receptor tyrosine kinase for colony-stimulating factor 1 and interleukin-34, playing a central role in the survival, proliferation and differentiation of mononuclear phagocytes, and linking tumor cells to the immune/mesenchymal microenvironment. The CSF1–CSF1R axis is a key driver of the immunosuppressive tumor microenvironment in TNBC, where high CSF1 expression by tumor cells recruits and polarizes tumor-associated macrophages (TAMs) toward a pro-tumorigenic M2-like phenotype [132]. These TAMs promote invasion, angiogenesis, CSC maintenance, and resistance to therapy, making CSF1R a central regulator of TNBC aggressiveness [133]. Elevated CSF1R expression in breast cancer tissues correlates with poor prognosis, increased TAM infiltration, and an immunosuppressive microenvironment [134]. In preclinical TNBC models, pharmacologic CSF1R inhibition reduces TAM abundance, suppresses tumor growth and metastasis, and enhances chemotherapy efficacy [132]. Additional studies indicate that CSF1R signaling extends beyond macrophages, influencing stromal and myeloid populations that support tumor progression [135]. Given these findings, CSF1R blockade—alone or in combination with chemotherapy or immunotherapy—represents a promising strategy for targeting the TNBC microenvironment [136]. However, because CSF1R is essential for normal myeloid homeostasis, therapeutic benefit will likely require biomarker-guided patient selection and rational combination approaches [132,134].

### 3.7. Ephrin Type B Receptor 3 (EPHB3)

EPHB3 is a member of the Eph receptor family of receptor tyrosine kinases that binds ephrin-B ligands in a contact-dependent manner, modulating cell–cell adhesion, migration, angiogenesis and developmental cell positioning. It shows consistent overexpression in TNBC across multiple transcriptomic datasets, including TCGA [137] and METABRIC [138,139], where its expression is enriched in basal-like subtypes and associated with proliferative and invasive signatures. Despite this reproducible omics-level signal, no functional studies have yet defined the role of EPHB3 in TNBC, distinguishing it from other Eph receptors (such as EPHB2 or EPHB6) that have already established mechanistic links to breast cancer progression. Though EPHB3 looks like a TNBC-specific candidate whose biological significance remains to be elucidated, it has been functionally implicated in several malignancies outside TNBC, with strongly context-dependent roles. In NSCLC, EPHB3 is overexpressed and promotes tumor cell growth, survival, migration and metastasis in a largely kinase-independent manner [140]. In contrast, a subsequent study in NSCLC showed that forced activation of EPHB3 kinase suppresses cell migration and metastatic seeding via a PP2A/RACK1/Akt signaling complex, revealing a tumor-suppressive pathway when ligand-induced signaling is restored [141]. In papillary thyroid cancer, EPHB3 acts as a tumor promoter, enhancing in vitro migration and in vivo metastasis through a kinase-dependent Vav2–Rho GTPase axis [142]. In colorectal cancer, several lines of evidence instead support a tumor-suppressor role: overexpression of EPHB3 in HT-29 colon cancer cells strengthens cell–cell contacts and suppresses tumor growth [99], and EPHB3 is frequently silenced in human colorectal cancer through “decommissioning” of a transcriptional enhancer [143]. Clinicopathological analyses further show that EPHB3 expression tends to be higher in early colorectal lesions and reduced in advanced tumors, with loss of EPHB3 correlating with poorer prognosis [144]. Recent comprehensive reviews of the Eph/ephrin system in cancer highlight EPHB3 as a prototypical example of a receptor whose activity can be either oncogenic or tumor-suppressive depending on ligand availability, phosphorylation state and tissue context [145].

### 3.8. Tribbles Pseudokinase 1 (TRIB1)

TRIB1 is a pseudokinase/adaptor protein that engages with ubiquitin ligases and signaling modules to regulate MAPK cascades, substrate degradation and cellular responses to stress or differentiation cues. TRIB1, a member of the Tribbles pseudokinase family, has emerged as a regulator of oncogenic pathways relevant to TNBC. TRIB1 is frequently overexpressed in breast cancer, where it promotes tumor progression through modulation of MAPK/ERK and NF-κB signaling [146]. Elevated TRIB1 expression has been associated with poor prognosis and increased tumor aggressiveness in breast cancer cohorts, including basal-like subtypes that overlap with TNBC [146]. Mechanistically, TRIB1 enhances survival and chemoresistance through stabilization of oncogenic proteins and suppression of apoptosis pathways [147]. In macrophages, TRIB1 is required for the differentiation of M2-like tumor-associated macrophages (TAMs) [148], suggesting that TRIB1-positive tumors may foster an immunosuppressive microenvironment—an established hallmark of TNBC. Furthermore, TRIB1 regulates inflammatory cytokine production and interacts with COP1, influencing degradation of transcription factors involved in tumor immune evasion [147]. Together, these findings indicate that TRIB1 contributes to TNBC biology, both at the cell level, by promoting survival and oncogenic signaling, and at the TME level, by supporting TAM-mediated immunosuppression. These dual functions position TRIB1 as a potential prognostic marker and a candidate therapeutic target in TNBC.

### 3.9. Ladinin-1 (LAD1)

LAD1 (Ladinin-1) is an anchoring-filament protein that resides in the basement membrane zone, contributing to epithelial–mesenchymal interface integrity and intercellular adhesion. It has recently been implicated in cancer progression through effects on cytoskeletal dynamics and cell motility. Quantitative proteomics identified LAD1 as a filamin-binding regulator of actin remodeling and reported its association with aggressive breast tumors [149]. Beyond breast cancer, LAD1 overexpression has been linked to poor clinical outcomes in multiple malignancies. In lung adenocarcinoma, high LAD1 expression promotes proliferation and migration and correlates with reduced overall survival [150]. In colorectal cancer, LAD1 upregulation is associated with metastatic potential and adverse prognosis, suggesting involvement in EMT and extracellular matrix remodeling [101]. More recently, LAD1 was shown to localize to actin-rich structures at invasive tumor fronts in oral squamous cell carcinoma, reinforcing its role in cytoskeletal reorganization during invasion [151]. Although functional studies of LAD1 in TNBC are currently lacking, its recurrent association with aggressiveness across several cancer types highlights LAD1 as a compelling candidate biomarker and potential mediator of invasion whose role in TNBC warrants focused investigation.

## 4. Metabolic Vulnerabilities in TNBC

Metabolic plasticity is a powerful tool for cancer cells to survive in extremely stressful conditions. Cancer cells can adapt to environmental stress with different strategies. According to recent studies on TNBC, the most frequent metabolic switches involve (i) enhanced glycolysis, (ii) enhanced oxidative phosphorylation, and (iii) fatty acid oxidation. Targeting proteins involved in the regulation of such relevant metabolic salvage pathways can offer good therapeutic approaches in difficult-to-treat and heterogeneous cancers like TNBC. As proved by Warburg and colleagues in 1927 [152], cancer cells rely on improved glycolysis even in normoxic conditions to increase the energetic metabolism required for fast proliferation. According to several reports, TNBC cells show increased dependency on glycolytic flux to proliferate and metastasize. This metabolic rewiring, driven prevalently by *TP53* or *Kras* mutations, *myc* amplification and, in general, dysregulated pro-proliferative pathways, causes increased expression of glycolytic enzymes (i.e., lactate dehydrogenase, LDH) and glucose transporters (i.e., GLUT).

Glucose transporter 1 (GLUT-1) is central in supporting this metabolic alteration; indeed, GLUT-1 alterations are frequent in cancers. In BC, high GLUT-1 expression correlates with increased proliferative activity, high histologic grade and drug resistance and is associated prevalently with basal-like carcinoma (ER-, PR-, HER-), the more represented subtype of TNBC [153]. In preclinical models of TNBC, inhibition of GLUT-1 using either small molecules such as STF-31, WZB-117 or BAY-876, or shRNA showed a marked anti-proliferative effect, supporting the possibility of targeting GLUT-1 for TNBC therapy [154,155,156,157]. Overexpression of glycolytic enzymes such as hexokinase 2 (HK2) and Lactate Dehydrogenase (LDH) promotes tumor growth and survival under hypoxic conditions [158]. HK2, an enzyme that catalyzes the first step of glycolysis, is upregulated in TNBC, and its upregulation is associated with enhanced energy production and resistance to pro-apoptotic signaling [159,160]. HK2 is anchored to the mitochondrial voltage-dependent anion channel (VDAC), and this interaction favors energy production and apoptosis escape by coupling glycolysis to ATP production. One possible strategy to target glucose metabolism in TNBC is to disrupt this interaction to reduce energy availability and successfully trigger pro-apoptotic pathways [161]. In preclinical models, direct targeting of HK2 has been attempted by using 2-deoxy-D-glucose (2-DG), a glucose analog that competitively inhibits HK2, causing ATP depletion and endoplasmic reticulum stress. The results reported by O’Neill et al. [162] showed that 2-DG reduces the viability of TNBC cells and enhances sensitivity to chemotherapeutics such as doxorubicin and paclitaxel. Ganapathy-Kanniappan et al. [163,164] have also shown in vivo marked inhibition of HK2 by using more specific small molecules like 3-bromopyruvate (3-BrPA) and lonidamine in xenograft models [165]. The data obtained showed significant tumor regression but also elevated systemic toxicity. HK2 expression can also be reduced by administering metformin, which acts through the AMPK pathway activation and activates p53 pro-apoptotic signaling [166].

Another glycolytic enzyme that is overexpressed in TNBC and is relevant to supporting tumor growth, invasion and immune escape is LDH, which catalyzes the conversion of pyruvate to lactate, regenerating the NAD^+^ required to sustain glycolysis even in hypoxic environments typical of solid tumors. Indeed, HIF-1α directly upregulates both HK2 and LDH transcription, enabling TNBC cells to survive and proliferate under oxygen deprivation [167]. Elevated LDH-A isoform expression in TNBC correlates with poor prognosis and enhances invasion and immune evasion, very likely by promoting an acidic tumor microenvironment [168,169,170,171]. Pharmacological inhibition of LDH using small molecules such as FX11 and GNE-140 has shown efficacy in preclinical TNBC models, reducing tumor growth and enhancing cell death [172,173]. Combination strategies integrating glycolytic inhibition with immune checkpoint inhibitors are being investigated to potentiate antitumor immunity and overcome metabolic resistance [174,175].

Glutamine addiction is another critical driver for metabolic plasticity observed in TNBC. Glutamine is the main extracellular source of carbon and nitrogen that not only fuels the tricarboxylic acid cycle (TCA) but also is involved in nucleotides synthesis and redox homeostasis. Glutamine transporters such as SNAT2/SLC38A2 and SLC7A5 are often overexpressed in TNBC cells [176,177]. Their blockade using specific inhibitors has been shown to reduce glutamine uptake and suppress tumor growth in in vitro and in vivo systems [178,179,180]. Another hallmark of glutamine addiction in TNBC is the overexpression of glutaminase 1 (GLS1), the enzyme that catalyzes the conversion of glutamine into glutamic acid, thus providing key precursors for the TCA cycle and antioxidant molecules and supporting ATP production [181,182]. Inhibition of GLS using small molecules like telaglenastat has proven effective in preclinical models by reducing energetic metabolism and cell proliferation and enhancing cancer cells’ sensitivity to immune checkpoints and standard chemotherapeutic agents [183,184].

Another relevant metabolic change often observed in cancer cells is aberrant lipid metabolism, which significantly contributes to cancer cells aggressiveness, metastasization and resistance to therapy [185,186,187]. Also, TNBC cells rely on de novo lipogenesis and fatty acid oxidation to sustain adaptation to nutrient and oxygen stress [188,189]. Overexpression of Fatty Acid SyNthetase (FASN) is a hallmark of TNBC and correlates with poor prognosis and therapeutic response [190,191]; therefore, FASN could be a valid target for targeted anti-metabolic TNBC therapy. In vitro inhibition of FASN using small molecules (i.e., TVB-2640 or C75) has been proven to be effective in reducing cell proliferation, in activating apoptosis and in sensitizing TNBC cells to combined therapy [192,193,194].

Fatty acid oxidation (FAO) is catalyzed by several enzymes; among them, Carnitine Palmitoyltransferase 1A (CPT1A) has a key role in mitochondrial FAO as a rate-limiting enzyme. CPT1A is often upregulated in TNBC, and its pharmacological inhibition by etomoxir or perhexiline has been attempted in preclinical models of TNBC, with good results in the inhibition of cell proliferation and tumor metastasis [188,195,196]. Another route to target lipid metabolism in cancer cells is the inhibition of Sterol Regulatory Element-Binding Proteins (SREBPs) which transcriptionally regulate lipid biosynthesis and are upregulated in TNBC [197]. Presently, there are no records of successful targeting of SREBPs in TNBC in vitro or in vivo models.

All in all, targeting metabolism in TNBC is a promising route for improved therapy, but systemic toxicity and off-target effect limit the use of this approach into the clinic, which requires further effort in improving targeting, selectivity and localization. Moreover, as shown by Cai et al. subsets of TNBC also exhibit metabolic plasticity [189], suggesting that combination strategies targeting multiple metabolic pathways may be required to achieve durable therapeutic responses. Therefore, besides targeted therapy, robust biomarkers are needed to guide patients’ stratification and improve personalized and targeting selection to optimize the risk/benefit ratio.

Main metabolic pathways altered in TNBC are summarized in Table 4.

## 5. Tumor Microenvironment and Immune-Modulating Factors

The tumor microenvironment (TME) is a complex ecosystem composed of epithelial cells, adipocytes, fibroblasts, immune cells, and soluble factors that collectively influence tumor progression and therapeutic response. In TNBC, the immune composition of the TME plays a decisive role in shaping disease behavior. TNBCs can be broadly divided into “hot” and “cold” tumors: hot tumors exhibit abundant immune infiltration and active antitumor immunity, whereas cold tumors are characterized by poor T-cell infiltration, defective antigen presentation, and enrichment in immunosuppressive cell types such as TAMs and myeloid-derived suppressor cells (MDSCs) [199]. These immune-excluded tumors typically show resistance to immune checkpoint inhibitors (ICIs), although therapeutic interventions may reprogram the immune context and promote conversion toward more inflamed phenotypes [200]. Recent studies have further stratified TNBC into four tumor immune microenvironment (TIME) subtypes: ID (immune-desert), MR (margin-restricted), SR (stroma-restricted), and FI (fully-inflamed), defined by distinct spatial patterns of CD8^+^ T-cell localization [201,202]. ID and MR tumors, both poorly infiltrated, display fibrosis and B7-H4 expression, correlating with poor prognosis. SR tumors show CD8^+^ T-cell accumulation restricted to the stroma, with IL-17^+^ cells, neutrophils, and stromal PD-L1/IDO1 expression creating an immunosuppressive environment. In contrast, FI tumors exhibit widespread infiltration of activated CD8^+^GzmB^+^ T cells, strong type I interferon signatures, and high checkpoint expression (PD-L1, LAG-3, TIM-3, TIGIT, CTLA-4), features that are associated with improved prognosis and sensitivity to immunotherapy [202].

### 5.1. Pharmacological Targeting of EMT in TNBC

The process known as epithelial-to-mesenchymal transition (EMT) endows epithelial cells with enhanced motility and invasive potential. Although this phenomenon is common across various malignancies, in TNBC EMT is particularly promoted by the activation of the NF-κB signaling pathway [203]. This transition is frequently associated with therapeutic resistance and metastatic competence, largely due to the regulation of transcription factors involved in oxidative stress responses and the production of reactive oxygen species (ROS) [204]. Several genes and molecular pathways have been identified as central regulators of EMT and thus represent promising therapeutic targets for the development of personalized strategies in TNBC [205]. Among these, inhibition of the receptor tyrosine kinase AXL has been shown to reduce cell migration and invasive behavior in mesenchymal TNBC cells, indicating that targeting AXL with R428 treatment may help regulate cell polarity [206]. Similarly, the Src/ABL inhibitor dasatinib induces epithelial polarization, sensitizing TNBC cells to paclitaxel, a conventional chemotherapeutical drug [207]. At the epigenetic level, pro-EMT and hypoxia-responsive genes are repressed using bromodomain and extra-terminal (BET) inhibitors, thereby limiting tumor progression and metastatic dissemination [208]. Moreover, epigenetic modification induced by histone deacetylase can be another interesting target: indeed, its inhibition by panobinostat (LBH589) in combination with salinomycin, an antibiotic effective against breast cancer stem cells, downregulates mesenchymal markers, leading to tumor regression in xenograft models [209]. More recently, synergistic effects were highlighted also with napabucasin, a STAT3 inhibitor, in combination with paclitaxel; in particular, napabucasin impairs mitochondrial function and cell growth in paclitaxel-resistant TNBC models [210]. Furthermore, suppression of EMT transcription factors such as SLUG and the inhibition of its interaction with LSD1 may serve as an adjuvant therapy [211]; more recently, a phase 1 proof-of-concept study demonstrated antitumor activity and the suppression of the overexpressed Lysine-Specific Demethylase-1 (LSD1) EMT marker using phenelzine, an LSD1 inhibitor, in combination with nab-paclitaxel [212]. Collectively, these findings highlight the therapeutic relevance of interfering with key EMT-related pathways, including NF-κB, STAT3, and epigenetic regulators, as a promising strategy to overcome phenotypic plasticity, metastasis, and drug resistance in TNBC.

### 5.2. Cancer-Associated Adipocytes as Mediators of Immune Evasion and Metabolic Crosstalk

Cancer-associated adipocytes (CAAs) constitute a significant number of adipocytes in the TME. They produce several inflammatory factors, among which are IL-1β, IL-6, IL-8, TNF-α, CCL2, leptin and free fatty acids [213]. CAAs are distinguished from mature adipocytes by their more elongated morphology, which tends to make them resemble fibroblasts, and by their irregular shape, their smaller size and the presence of small lipid droplets [214]. It has been observed that the production of IL-8 by CAAs causes a remodeling of the TME by suppressing CD4+ T and CD8+ T immune cell infiltration, but also by upregulating PD-L1 expression in TNBC. In this case, a promising therapeutic strategy is to target the IL-8 production pathway in association with immunotherapy by blocking the PD-1 pathway [215]. Another immunosuppressive strategy is the production by CAAs of CCL2, a cytokine that recalls monocytes and macrophages in the tissue, differentiating them into Myeloid-Derived Suppressor Cells (MDSCs) and M2 macrophages. A recently developed strategy to reverse this behavior exploits CCL2-targeted lipid-protamine-DNA nanoparticles, capable of suppressing CAAs, which increase T-cell infiltration and decrease the population of immunosuppressive M2 macrophages and MDSCs [216]. The role of adipocytes adjacent to the tumor, however, is not yet fully explored; it has only recently been observed that CAAs and tumor cells could create gap junctions, through which an exchange of metabolites becomes possible, favoring tumor support and development [217]. CAAs can therefore not only release free fatty acids into the TME, but can also activate lipolysis processes to generate metabolites to be exchanged directly through gap junctions established with tumor cells. This discovery paves the way for the development of molecules and drugs that can re-modulate the expression of cell adhesion molecules. A key mediator discovered in the regulation of metabolic interactions between CAAs and tumor cells is ANGioPoieTin-Like 4 (ANGPTL4), a factor expressed on TNBC cells, which stimulates adipocyte-driven glycolysis and metastasis [218]. A new treatment technology that has been under development is the transplantation of generated adipocytes placed alongside cancer cells or xenografts, capable of upregulating UnCoupling Protein 1 (UCP1) with consequent suppression of tumor growth [219].

### 5.3. Cancer-Associated Fibroblasts as Drivers of Fibrosis, Hypoxia and Drug Resistance

In the TME it is often possible to recognize a cellular component of cancer-associated fibroblasts that are called CAFs. Their origin is not fully elucidated: CAFs might derive from adipocytes or from mesenchymal stem cells [220], but the most highly quoted hypothesis is that they derive from normal fibroblasts. CAFs display a more active phenotype than normal fibroblasts, such as increased proliferation, migration, invasion, tumorigenicity, and chemoresistance [221]. CAFs were associated with the infiltration of CD163+ macrophages and lymphatic metastasis and may be potential prognostic predictors of TNBC [220]. A peculiar characteristic of CAFs associated with TNBC is the increased deposition of collagen in the extracellular matrix, which is associated with a higher risk of developing metastases. This mechanism is mediated by TGF-β; in fact, by using one of its antagonists, pirfenidone, a reduction in the proliferation and production of collagen is obtained in in vitro models. Moreover, reduced tumor growth and lung metastasis formation were observed in in vivo models with a synergistic combination of pirfenidone with doxorubicin [222]. Using xenograft models, attempts have also been made to use specific inhibitors for Fibroblast Activation Protein (FAP), a membrane protein expressed in CAFs, which has a gelatinolytic activity; promising antitumor results were obtained by inhibiting this protein [223]. One of the factors that induces resistance to therapies is hypoxia, and CAFs also have a role in the regulation of this pathway in TNBC: in particular, CAFs present in the hypoxic portion secrete very high levels of Colony-Stimulating Factor 3 (CSF3), a factor that activates the invasive behavior of TNBC cells, through PhosphoGlucoMutase 2-Like 1 (PGM2L1) [224]. This discovery highlights a new potential target for developing therapies especially in relapsing cases, with residual disease induced by hypoxic resistance. Extremely interesting new technologies for developing therapeutic approaches rely on formulations based on nanoparticles co-modified with anisamide, a drug carrier, and CAF cell membranes, to load tetrandrine, a calcium channel blocker, as a CAF nano-regulator, which can precisely target and modulate CAFs [225].

### 5.4. Targeting Immune Cells in TME

TNBC is the BC subtype most suitable for immunotherapies; indeed, its TME is characterized by a high number of macrophages and cytotoxic T cells, compared to other breast cancer subtypes [226]. Furthermore, recent studies have demonstrated the high heterogeneity of TNBC, dividing this class into five immune subtypes—(i) T/B-cell/IFN High, (ii) IFN/Class Switch Recombination (CSR) High, (iii) CSR High, (iv) TGF-b High, and (v) Immune Low—based on four distinct expression signatures and derived from four core coexpression modules that capture distinct immune components and signaling programs (T/B-cell, IFN, TGF-b, and CSR) [227]. A highly expressed marker in TNBC is PD-L1: for this reason, PD-L1 antibodies, as monotherapy or a part of combined therapies, have been applied in multiple clinical trials, confirming that the combined therapy yields synergistic effects. ICIs Atezolizumab (a PD-L1 antibody) combined with paclitaxel has been approved as therapy for PD-L1–positive TNBC [228]. However, the rationale of the combination and successful application of anti-PD-L1 therapy relies on a comprehensive understanding of PD-L1 biology in tumor cells. It has been demonstrated that anti-VEGFR2 therapy is necessary to make TNBC cells more sensitive to ICIs; the combination of apatinib with camrelizumab and chemotherapy promotes CD8+ T-cell infiltration and activation within the TME, alongside anti-angiogenic-induced PD-L1 upregulation [229]. Although ICIs have proven to be a very promising strategy, the relapse rate of TNBC remains high; for this reason, technologies have been developed that allow the transfer of engineered T-cells such as chimeric antigen receptor T (CAR-T) cells. To make this technique effective, it is necessary to find antigens that have a higher level of expression in the tumor than in healthy tissues; however, it has been seen that CAR-T cells have a modest efficacy in solid tumors due to high cellular heterogeneity. For this purpose, it has been proposed to develop CAR-T cells capable of recognizing multiple markers simultaneously; optimizing CAR-T cells to recognize the CD276 and CSPG4 markers, encouraging results were obtained on TNBC Patient-Derived Xenograft (PDX) models [230]. Another interesting approach to increase the effectiveness of CAR-T treatment is the combination with radiotherapy: exposure to radiation favors the infiltration of T and NK cells, but also the expression of ICAM-1 in TNBC cells. EGFR-targeted CAR-T therapy combined with radiotherapy inhibits tumor progression and prolongs survival [231]. A similar technology is also being developed for CAR-M (where M stands for Marophages): currently, there are very encouraging data for HER2+ tumors with limited sensitivity to anti-PD1 monotherapy [232], and this seems to be an interesting path to explore and test also for TNBC. The immune system, however, can recognize cancer neoantigens that are mutant proteins/amino acid sequences. For this reason, neoantigen vaccines, that can induce neoantigen-specific CD8 and CD4 T cell responses and antitumor immunity, as well as neoantigen DNA vaccines, have been developed. DNA vaccination is a direct administration of recombinant DNA, and, in a phase 1 clinical trial, it was demonstrated that DNA vaccines are safe and well-tolerated in patients with persistent TNBC following neoadjuvant chemotherapy, with no significant adverse events. The neoantigen DNA vaccines were able to induce neoantigen-specific immune responses, with evidence of improved RFS [233].

### 5.5. Soluble Factors Released in the TME as Modulators of TNBC Target Therapy

Cytokines and chemokines are among the main soluble factors present in the TME, and they are fundamental players in the modulation of the immune response. However, immune modulation is not exclusive to these factors, and also proteins of the extracellular matrix, metabolites and exosomes can be potential therapeutic targets. Starting from transcriptomic data, it has been observed that, in TNBC, there is an upregulation of genes coding for the collagen family, while those of the integrin family are downregulated [234]. In matrix remodeling, metalloproteinases certainly have a decisive role: indeed, Matric Metalloprotease-14 (MMP14) and DDR2 have been proposed as diagnostic markers for metastatic TNBC [235]. An interesting new target for defining the metastatic capacity of TNBC is O-glycosylation: different highly O-glycosylated protein-coding genes, such as *mmp9*, *ecm1*, and *ank2*, were upregulated in a mouse model expressing the sugar Tn antigen (GalNAc-O-Thr/Ser), an antigen expressed in many adenocarcinomas and absent in healthy tissues. Incomplete O-glycosylation leads to the expression of an altered form of the Tn antigen, which might regulate their activity or interaction with different molecules and promote cancer development and immunoregulation. The role of O-glycans in the identified molecules remains to be elucidated [236]. Therefore, soluble factors cannot currently be considered effective therapeutic targets but could rather be used as diagnostic or prognostic markers to monitor the progression of the disease. In this context, extracellular vesicles are also considered promising diagnostic biomarkers and novel drug delivery systems among the prospects for immunotherapy in TNBC [237].

## 6. Targeted Radiotherapy in TNBC

Current guidelines recommend the use of radiotherapy following surgical resection, with the aim of eliminating residual tumor cells and reducing the risk of recurrence while minimizing toxicity as much as possible by the adoption of the optimal treatment plan [238]. Current studies are also developing new approaches to optimize radiotherapy outcomes using radiosensitizer-enhancers and nano-radiotherapy. Radiosensitizer-enhanced radiotherapy is an approach that uses drugs capable of making tumor cells more sensitive to ionizing radiation [6]. From this perspective, nanomaterials such as AGuIX nanoparticles, polysiloxane nanoparticles containing the metal gadolinium (Gd) as a high atomic number element, are also being developed. In particular, it has been demonstrated that AGuIX nanoparticles may be used as effective radiosensitizers in TNBC, promoting not just DNA damage and apoptosis, but also ferroptosis by the NRF2-GSH-GPX4-dependent pathway after irradiation [239]. In an attempt to make these applications as direct as possible on molecular targets, a recent in vitro study has demonstrated that targeting Elongin B in TNBC holds great promise for improving radiotherapy outcomes: Elongin B’s role has been discovered to be pivotal in regulating mitochondrial function via modulating mtDNA expression and the activities of respiratory chain complexes. However, specific inhibitors for Elongin B are not yet known [240]. Gemcitabine also has a sensitizer effect; to make the treatment more effective, the drug has been combined with a radionuclide in ^131^I-labelled polydopamine encapsulated gold nanoparticles within a hydrogel formed from oxidized glucan and chitosan hydrochloride [241].

In the context of developing radiotherapy approaches that are directly targeted to specific molecular determinants, applications in TNBC remain extremely limited. To date, one of the few examples reported in the literature is the study by Sankaranarayanan et al. [242], which investigated a theranostic strategy in a TNBC xenograft mouse model through the radiolabeling of a close derivative of the PARP inhibitor olaparib (PARPi-01) with the Auger electron emitters ^123/125^I. Auger electron emitters release cascades of low-energy electrons upon radioactive decay, inducing highly localized DNA damage. Owing to the nuclear localization of PARP1, PARP inhibitors represent particularly suitable backbone molecules for radiolabeling with Auger emitters. Among these, ^125^I is one of the most efficient Auger emitters, capable of releasing up to 23 Auger electrons per decay, and is commercially available, making it a promising candidate for highly localized, molecularly targeted radiotherapy approaches in TNBC.

Overall, it is clear that a multimodal approach, which attacks the tumor from multiple fronts, is the most promising strategy, and targeted radiotherapy approaches deserve dedicated investigations also for TNBC treatment, especially with the goal of personalizing therapy as much as possible, to minimize the risk of recurrence and therapy-related side effects.

## 7. Conclusions

The aim of this review is to present a broad overview of possible new targets for the treatment of TNBC. While well-defined and clinically applicable guidelines have been successfully established for breast cancers characterized by the expression of specific receptors, this is not the case for TNBC. This subtype is primarily defined by the absence of known receptors, and to date, no specific protein target has been clearly identified as a hallmark that could be exploited therapeutically. The purpose of this review is therefore to explore, primarily through the analysis of omics databases, potential protein targets expressed either at the cellular level or within the tumor microenvironment that could be defined as molecular signatures characteristic of TNBC. This approach aims to lay the groundwork for future studies focused on the development of molecules capable of counteracting tumor growth. Ultimately, the objective is to move from a classification based on exclusion, defining TNBC as a tumor that does not belong to other breast cancer subtypes, toward a more precise characterization based on the expression of specific targets. Clearly, this review cannot address this challenge in an exhaustive manner, as the multifactorial nature of TNBC is extremely complex, and its classification is not univocal but rather depends on phenotypic features, genetic alterations, and interactions with the diverse components of the tumor microenvironment. Further in situ transcriptomic and proteomic studies will be required to achieve a more refined subtyping of TNBC and, consequently, to better define potential therapeutic strategies that can be evaluated in clinical trials.

## Figures and Tables

**Table 1:** List of current and prematurely terminated clinical trials.

Trial	Investigational Drug	Target	Year	Phase	State	Results	Reference
FASCINATE-N (ClinicalTrials.gov ID NCT05582499)	New targeted drugs	PARPi, Trop-2, Antibody–drug conjugates, CDK 4/6 inhibitors, PD-L1 mAb, HER2 inhibitor, anti-angiogenic agents	2022–2028 (estimated)	II	Recruiting	N.A.	[23]
TROPION-Breast05 (ClinicalTrials.gov ID: NCT06103864)	Durvalumab (Imfinzi)	Anti-PD-L1	2023–2029 (estimated)	III	Recruiting	N.A.	[25]
NCT03170960	Atezolizumab (Tecentriq)	Anti-PD-L1	2017–2027 (estimated)	I	Active, not recruiting	N.A.	N.A.
PAveMenT (ClinicalTrials.gov ID: NCT04360941)	Avelumab (Bavencio)	Anti-PD-L1	2026–2026 (estimated)	I	Recruiting	N.A.	N.A.
NCT02936102	FAZ053	Anti-PD-L1	2016–2024	I	Terminated	N.A.	N.A.
NCT04916002	Cemiplimab	Anti-PD-1	2021–2024	II	Terminated	N.A.	N.A.
NCT03549000	PDR001	Anti-PD-1	2018–2022	I	Terminated	N.A.	N.A.
PARTNER (ClinicalTrials.gov ID NCT03150576)	Olaparib	PARP	2016–2034 (estimated)	II-III	Recruiting	N.A.	[26,27,28]
NCT05933265	LP-184	PARP	2023–2025 (estimated)	I-II	Recruiting	N.A.	[29]
NCT03875313	Telaglenastat (CB-839), Talazoparib	PARP	2019–2022	I-II	Terminated	N.A.	N.A.
NCT03801369	Olaparib	PARP	2018–2024	II	Terminated	N.A.	[30]
NCT04916002	Vidutolimod, Cemiplimab	PARP	2021–2024	II	Terminated	N.A.	N.A.
NCT05252390	NUV-868, Olaparib, Enzalutamide	PARP	2022–2024	I	Terminated	N.A.	N.A.
NCT02419495	Selinexor (KPT-330)	PARP	2015–2024	I	Terminated	N.A.	N.A.
NCT02627430	Talazoparib, Hsp90 Inhibitor AT13387	PARP	2016–2019	I	Withdrawn	N.A.	N.A.
NCT07046455	Sacituzumab Govitecan (SG) and PET Probes	Trop-2	2025–2027 (estimated)	N.A.	Recruiting	N.A.	N.A.
FUTURE2.0 (ClinicalTrials.gov ID NCT05749588)	SHR-A1811, TROP2 ADC, BP102	Trop-2	2023–2026 (estimated)	II	Recruiting	N.A.	N.A.
BALISTA (ClinicalTrials.gov ID NCT06793332)	IvoneScimab, Trop2 ADC	Trop-2	2024–2027 (estimated)	II	Recruiting	N.A.	[31,32,33,34]
NCT06851299	Trop2-ADC monotherapy	Trop-2	2025–2028 (estimated)	II	Recruiting	N.A.	N.A.
MK-2870-011/ TroFuse-011 (ClinicalTrials.gov ID NCT06841354)	Sacituzumab Tirumotecan (Sac-TMT, MK-2870) and Pembrolizumab (MK-3475)	Trop-2	2025–2030 (estimated)	III	Recruiting	N.A.	N.A.
NCT06878625	Trop-2 ADC Combination Therapy	Trop-2	2024–2027 (estimated)	II	Recruiting	N.A.	N.A.
NCT06649331	Anti Trop-2 antibody-conjugated drugs (ADCs)	Trop-2	2024–2027 (estimated)	II	Recruiting	N.A.	N.A.
TROPION-DM (ClinicalTrials.gov ID NCT06974604)	Dexamethasone	Trop-2	2025–2029 (estimated)	II	Recruiting	N.A.	N.A.
NCT06103864	Dato-DXd	Trop-2	2023–2029 (estimated)	III	Recruiting	N.A.	[25]
NCT03901469	ZEN003694, Talazoparib	Trop-2	2019–2024	II	Terminated	N.A.	[35]
ASPRIA (ClinicalTrials.gov ID NCT04434040)	Sacituzumab govitecan, Atezolizumab	Trop-2	2020–2027 (estimated)	II	Recruiting	N.A.	N.A.
NeoSACT (ClinicalTrials.gov ID: NCT04877821)	Anlotinib (FOCUS V)	Anti-angiogenic agent	2021–2025	II	Active, not recruiting	pCR: 69% MRD: 86.2% 2y EFS: 92.4%	[36]
NCT06724263	B1962	Anti-angiogenic agent	2024–2026 (estimated)	II	Not yet recruiting	N.A.	N.A.
NCT06189209	Tenalisib	PI3K inhibitor	2024–2026 (estimated)	II	Recruiting	N.A.	N.A.
NCT03218826	AZD8186	PI3K inhibitor	2024–2026 (estimated)	I	Active, not recruiting	MTD: NR Anemia: 57% Diarrhea: 43% Fatigue: 43%	[37]
SABINA (ClinicalTrials.gov ID NCT05810870)	MEN1611	PI3K inhibitor	2023–2027 (estimated)	II	Recruiting	N.A.	N.A.
BCTOP-T-M03 (ClinicalTrials.gov ID: NCT05954442)	Everolimus (Afinitor)	PI3K inhibitor	2023–2026 (estimated)	III	Recruiting	N.A.	N.A.
NCT01918306	GDC-0941	PI3K inhibitor	2013–2015	I-II	Terminated	N.A.	N.A.
NCT02457910	Taselisib	PI3K inhibitor	2015–2022	I-II	Terminated		N.A.
NCT04216472	Alpelisib	PI3K inhibitor	2020–2025	II	Terminated	N.A.	N.A.
NCT02476955	ARQ-092	PI3K inhibitor	2015–2019	I	Terminated	N.A.	N.A.
NCT03090165	Bicalutamide (Casodex)	AR inhibitor	2018–2025 (estimated)	I-II	Recruiting	N.A.	N.A.
NCT07016399	Darolutamide (Nubeqa)	AR inhibitor	2025–2033 (estimated)	II	Recruiting	N.A.	N.A.
CAREGIVER (ClinicalTrials.gov ID: NCT05067530)	Palbociclib (Ibrance)	CDK 4/6 inhibitor	2022–2026 (estimated)	II	Not yet recruiting	N.A.	N.A.
CHARGE (ClinicalTrials.gov ID: NCT04315233)	Ribociclib	CDK 4/6 inhibitor	2021–2026 (estimated)	I	Recruiting	N.A.	N.A.
NCT02978716	Trilaciclib	CDK 4/6 inhibitor	2017–2020	II	Terminated	See publication	[38,39]
NCT05113966	Trilaciclib	CDK 4/6 inhibitor	2021–2024	II	Terminated	PFS: 4.1 months ORR: 23.3% CBR: 46.7% DoR: 8.8 months OS: 15.9%	N.A.
NCT03519178	PF-06873600	CDK 4/6 inhibitor	2018–2024	I-II	Terminated		N.A.
NCT06264921	NKT3447	CDK 4/6 Inhibitor	2024–2025	I	Terminated	N.A.	N.A.

N.A. = not available. pCR = pathological complete response. MRD = minimal residual disease. mtd = maximum tolerated dose. NR = not reached. EFS = event-free survival. PFS = progression-free survival. ORR = overall response rate. CBR = clinical benefit rate. DoR = duration of response. OS = overall survival.

**Table 2:** New possible targets identified by omics with the Elicit AI research assistant (details in the text). UniProt link https://www.uniprot.org/uniprotkb/P04818/entry (accessed on the 15 December 2025).

Gene Symbol	Protein Name	UniProt ID
GGH	Gamma-glutamyl hydrolase	Q92820 (https://www.uniprot.org/uniprotkb/Q92820/entry (accessed on 31 December 2025))
TYMS	Thymidylate synthase	P04818 (https://www.uniprot.org/uniprotkb/P04818/entry (accessed on 31 December 2025))
PTK6	Protein-tyrosine kinase 6 (BRK)	Q13882 (https://www.uniprot.org/uniprotkb/Q13882/entry (accessed on 31 December 2025))
TOP1MT	DNA topoisomerase I, mitochondrial	Q969P6 (https://www.uniprot.org/uniprotkb/Q969P6/entry (accessed on 31 December 2025))
SMO	Smoothened receptor	Q99835 (https://www.uniprot.org/uniprotkb/Q99835/entry (accessed on 31 December 2025))
CSF1R	Colony-stimulating factor 1 receptor	P07333 (https://www.uniprot.org/uniprotkb/P07333/entry (accessed on 31 December 2025))
EPHB3	Ephrin type-B receptor 3	P54753 (https://www.uniprot.org/uniprotkb/P54753/entry (accessed on 31 December 2025))
TRIB1	Tribbles pseudokinase 1	Q96RU8 (https://www.uniprot.org/uniprotkb/Q96RU8/entry (accessed on 31 December 2025))
LAD1	Ladinin-1	O00515 (https://www.uniprot.org/uniprotkb/O00515/entry (accessed on 31 December 2025))

**Table 3:** Summary of role and evidence of potential targets derived from omics.

Protein	Role in Cancer Pathogenesis	Evidence in TNBC/Relevance	References
SMO (Smoothened)	Aberrant activation of SMO promotes proliferation, invasion, stem-cell-like traits and therapy resistance in various cancers.	SMO (and GLI1) expression correlates with higher grade, node positivity, poorer prognosis.	[96,97]
CSF1R (Colony-stimulating factor 1 receptor)	Promotes tumor-associated macrophage (TAM) support, immune evasion, angiogenesis and metastatic spread.	High CSF1R expression has been associated with inferior survival, and preclinical models show CSF1R inhibition reduces brain metastasis in TNBC.	[98]
EPHB3 (Ephrin type-B receptor 3)	Dysregulation can promote invasion/metastasis in cancers.	In TNBC, integrative genomic analyses have flagged EPHB3 as a hyperactivated gene and a potential target.	[99]
TRIB1 (Tribbles pseudokinase 1)	Overexpression correlates with poor prognosis; promotes resistance to therapy.	In breast cancer, elevated TRIB1 correlates with worse survival.	[100]
LAD1 (Ladinin-1)	Overexpression has been associated with more aggressive phenotypes in various cancers (breast, lung).	Higher LAD1 links to increased migration/metastatic potential; genomics in TNBC flag LAD1 as potential target.	[101,102]

**Table 4:** Altered metabolic pathways in TNBC.

Altered Metabolic Pathway	Main Molecular Targets	Role in TNBC	Targeting Strategies	Reference
Enhanced glycolysis (Warburg effect)	GLUT-1	Increased glucose uptake; high proliferative index, high histological grade, drug resistance and basal-like phenotype	STF-31, WZB-117, BAY-876, shRNA	[154,155,156,157]
	HK2	Promotes ATP production and apoptosis resistance via interaction with VDAC	2-deoxy-D-glucose (2-DG), 3-bromopyruvate, lonidamine, metformin	[162,165,166,198]
	LDH (LDH-A isoform)	Sustains glycolysis under hypoxia; promotes invasion, immune evasion and acidic tumor microenvironment	FX11, GNE-140	[172,173]
Hypoxia-driven metabolic regulation	HIF-1α	Transcriptionally upregulates HK2 and LDH, enabling survival under oxygen deprivation	Indirect targeting through glycolytic inhibition	[74,167]
Glutamine addiction	Glutamine transporters (SNAT2/SLC38A2, SLC7A5)	Increased glutamine uptake, increased TCA cycle, nucleotide biosynthesis and redox homeostasis	Specific transporter inhibitors	[176,177,180]
	GLS1	Fuels TCA cycle, ATP production and antioxidant defenses	Telaglenastat	[182,184]
De novo lipogenesis	FASN	Supports tumor growth, aggressiveness and therapy resistance; overexpression correlates with poor prognosis	TVB-2640, C75	[193,194]
Fatty acid oxidation (FAO)	CPT1A	Promotes adaptation to nutrient and oxygen stress	Etomoxir, perhexiline	[195,196]
Lipid metabolism transcriptional control	SREBPs	Master regulators of lipid biosynthesis; frequently upregulated in TNBC	No effective validated inhibitors	[191,197]
